# Culicidae-centric metabarcoding through targeted use of D2 ribosomal DNA primers

**DOI:** 10.7717/peerj.9057

**Published:** 2020-06-03

**Authors:** Pedro M. Pedro, Jandui Amorim, Martha V.R. Rojas, Ivy Luizi Sá, Allan Kardec Ribeiro Galardo, Noel Fernandes Santos Neto, Dario Pires de Carvalho, Kaio Augusto Nabas Ribeiro, Maria Tereza Pepe Razzolini, Maria Anice Mureb Sallum

**Affiliations:** 1 Departamento de Epidemiologia, Faculdade de Saúde Pública, Universidade de São Paulo, São Paulo, SP, Brazil; 2 Biomonitoring and Sustainability, IPE—Institute for Ecological Research, Nazaré Paulista, SP, Brazil; 3 IEPA—Instituto de Pesquisas Cientificas e Tecnológicas do Estado do Amapá, Macapá, AP, Brazil; 4 FUNDUNESP—Fundação para o Desenvolvimento da UNESP, São Paulo, SP, Brazil; 5 Santo Antônio Energia, Porto Velho, Brazil; 6 Departamento de Saúde Ambiental, Faculdade de Saúde Pública, Universidade de São Paulo, São Paulo, SP, Brazil

**Keywords:** Mosquito monitoring, Metabarcoding, D2 expansion segment, Abundance estimates, Non-target taxa

## Abstract

A practical limitation to many metabarcoding initiatives is that sampling methods tend to collect many non-target taxa, which become “amplicon noise” that can saturate Next Generation Sequencing results and lead to both financial and resource inefficiencies. An available molecular tool that can significantly decrease these non-target amplicons and decrease the need for pre-DNA-extraction sorting of bycatch is the design of PCR primers tailored to the taxa under investigation. We assessed whether the D2 extension segment of the 28S ribosomal operon can limit this shortcoming within the context of mosquito (Culicidae) monitoring. We designed PCR primers that are fully conserved across mosquitos and exclude from amplification most other taxa likely to be collected with current sampling apparatuses. We show that, given enough sequencing depth, D2 is an effective marker for the detection of mosquito sequences within mock genomic DNA pools. As few as 3,050 quality-filtered Illumina reads were able to recover all 17 species in a bulk pool containing as little as 0.2% of constituent DNA from single taxa. We also mixed these mosquito DNA pools with high concentrations of non-Culicidae bycatch DNA and show that the component mosquito species are generally still recoverable and faithful to their original relative frequencies. Finally, we show that there is little loss of fidelity in abundance parameters when pools from degraded DNA samples were sequenced using the D2 primers.

## Introduction

The measurement of presence, absence and abundance of biological entities, broadly defined as biomonitoring, is recognized as an important component of environmental assessments. For example, the detection within freshwater samples of certain proportions of microorganisms (e.g., Qureshi, 2003) and macroorganisms (e.g.,  Weijters et al., 2009) are known to be reliable proxies for the overall health of aquatic systems. Biomonitoring can also provide early detection of target species that can become crop pests, disease vectors or otherwise impact human wellbeing (e.g.,  Kogan, 1998; Walton et al., 1999). However, biomonitoring’s usefulness and applicability has been limited by its reliance on often-difficult taxonomic diagnoses (many times of animals of disparate size and of vastly varying life stages) and by the sheer quantity of individuals needed to produce statistically sound estimates within the context of broad ecological questions, such as consequences of climate alterations (Pereira & David Cooper, 2006).

The development of amplicon-based biomonitoring protocols within the framework of next-generation DNA sequencing (NGS), known as metabarcoding, now often obviates the identification bottleneck (through the use of “reference libraries” that link sequences to known species; Ratnasingham & Hebert, 2007) and is compatible with the high-throughput scaling needed for reliability and replicability (potentially yielding hundreds of gigabases of data at a reasonable cost). Thus, when protocols can be designed that selectively sample the target taxa, wet-lab sorting and other traditionally tedious impediments are no longer significant issues, and the emphasis is now on the optimization of the experimental design, including DNA extraction, PCR optimization, read analysis, and statistical treatments (Diaz-Real et al., 2015; Krehenwinkel et al., 2017; Macher et al., 2018).

Of special consideration to biomonitoring initiatives is that resulting datasets reflect not only diagnostic presence/absence parameters, but also yield a relatively faithful estimate of all the organisms sampled, if not the relative amounts of tissue/cells processed. This is an often difficult technical step (Diaz-Real et al., 2015; Krehenwinkel et al., 2017). In this regard, metabarcoding protocols tend to adopt either of two analytical strategies: (a) accept a level of *post hoc* sample processing (e.g., size-sorting taxa so that very large organisms do not overwhelm the sequencer signals at the expense of smaller taxa (Elbrecht, Peinert & Leese, 2017) or (b) tailor the molecular protocols to include only a taxonomic subset of the entire assemblage sampled, generally through primer design (De Barba et al., 2014). The former strategy is most relevant for broad-based ecological questions where the goal is a generalized reflection of the sampled habitat. These initiatives will generally use highly universal (or highly degenerate) primer sets, such that as much biodiversity is registered. However, they may lose substantial signal from several sources of noise, including disparate organism size (even after sorting) and varying amenabilities of specimens to DNA extraction protocols (Elbrecht, Peinert & Leese, 2017).

The second metabarcoding strategy utilizes the molecular “cherry-picking” of one or more taxonomic groups from the subset of the sampled organisms, thus losing much of the aggregate biodiversity data, but yielding robust estimates from a dataset free of taxonomic noise. Here, the breadth of the biodiversity output is delimited by the universality (or lack of) of the primers used to yield sequence reads from the target groups (Prigigallo et al., 2015; Reeves et al., 2018). Generally, organisms within these subsets of interest are phylogenetically proximal, will share similar DNA extraction parameters and be of similar biomass, thus requiring little pre-DNA-extraction sorting. For both its statistically robust estimates of the subset from the collected assemblage and the minimal amount of time and resources invested in wet-lab manipulations and/or sorting, this may be a more efficient monitoring strategy for some questions and in some contexts.

The family Culicidae is comprised predominantly of hematophagous species, a great portion of which are of epidemiological relevance to human and domestic animals. It thus offers a practical case with a valid question that targets family-level taxa. Mosquitos are especially amenable to a metabarcoding pipeline of pooled samples because there is less than a one-order-of-magnitude difference between the largest and smallest representatives and they vary very little in scleritome resistance. They thus represent a case where no special care must be taken to lyse all organisms equally. Of practical relevance is that current commercial attractant traps now allow capture on the scale of tens of thousands of individuals (Kline, 2002; Mann, Kaufman & Butler, 2009).

The few metabarcoding efforts in mosquito surveyance have used mitochondrial DNA markers. Cytochrome oxidase I has been the principal focus, and generally recovers large percentages of species in mock assemblages. The sequence abundance (i.e., biomass estimate), however, has been shown to be biased by the uneven phylogenetic affinity of the primers used (Batovska et al., 2018). Because at least one of the commonly used CO1 primers anneals to protein-coding regions, third-codon substitutions are common, even within species, which, depending on its proximity to the 3′-end of the primer, will lead to substantial amplification bias (Clarke et al., 2014; Deagle et al., 2014). Conversely, the highly conserved mitochondrial 16S primers used by Schneider et al. (2016) yielded a very high proportion of non-target sequences, in addition to the target culicids, resulting in substantial data loss and added cost.

Current technologies allow unprecedented quantities of culicids to be captured and monitored in a systematic format (using standardized odor baits, trap placement and duration of deployment; Sá & Sallum, 2013), but still sample a significant proportion of non-target insects (Pucci, Lynch & Keiper, 2003). There thus exists a challenge in estimating accurate frequencies of different species (in light of primer bias) whist also not swamping sequencing output with taxa of no surveillance value.

Here, we hypothesize that the D2 extension segment of 28S ribosomal DNA better fits the requirements described above for Culicidae metabarcoding. The D2, like CO1, is present in hundreds of copies per cell and is relatively well represented phylogenetically in the public nucleotide databases (Beebe, 2018). Priming sites are known to be conserved adjacent to this region and it has been shown to be as informative as mitochondrial markers to infer interspecific divergence (Krzywinski, Wilkerson & Besansky, 2001; Sallum et al., 2002). Moreover, the non-coding nature of D2 means that homoplasy is rare and thus distant taxa can be excluded from amplification through taxa-specific primer design at nucleotide positions present solely in the target species.

Specifically, we explore the utility of Culicidae-specific D2 primers to realistic conditions encountered in mosquito surveillance efforts. We initially compare the expected efficiency of previously used metabarcoding primers to those designed herein by evaluating their taxonomic coverage across mosquitoes and taxonomic exclusivity to Culicidae. We subsequently evaluate the faithfulness of NGS read outputs from pools of known volumes of mosquito template DNA following PCR with the D2 primers. We also verify the *in silico* expectation that this fidelity is maintained when mosquito pools are mixed with non-Culicidae DNA bycatch. Finally, we test the reproducibility of results between different amplifications of the same DNA pools and explore the impact of DNA degradation on estimates.

## Methods and Materials

### Testing primer coverage for mosquito metabarcoding

We mined GenBank (15/11/2018) for potential primer sequences that would maximize recovery of mosquito amplicons without bias and maximally exclude non-culicid insects. Initially, we selected only GenBank entries with at least a 30 bp on either side of the putative oligo to avoid database submissions that might also include original amplification-primer sequences.

From the available sequences, we identified a primer set that produced an amplicon from the D2 segment of the 28S operon. The forward primer (Mozzie.D2.Uni.F; 5′-AAGCACTCTGAATAGAGAGTC-3′) was designed at nucleotide position 67 and the reverse (Mozzie.D2.Uni.R; 5′-TGGTCCGTGTTTCAAGAC-3′) at nucleotide position 489 of the *Aedes aegypti* ribosomal DNA locus (GenBank accession no. MG242540).

We next tested binding site variation and the taxonomic resolution of Mozzie.D2.Uni.F and Mozzie.D2.Uni.R with only those GenBank sequences that possessed both primers (sequences initially used for primer design did not necessarily contain both priming sites) using the *ecopcr* module of Obitools (v1.2.13) (Boyer et al., 2016). For this analysis, we did not use the criterion for a 30 bp flank above because we sought primarily data on taxonomic resolution of the resultant amplicons.

We then compared these D2 results with primer sets previously used in mosquito metabarcoding studies. These included two sets from mitochondrial 16S and two from CO1 (Schneider et al., 2016; Talaga et al., 2017; Batovska et al., 2018; Krol et al., 2019).

We compared the primer taxonomic coverage and estimated taxonomic resolution of the resultant amplicons in culicids and non-culicids. The latter taxa were divided into separate groups based on our own experience of the bycatch content in commonly used mosquito traps and on relevant literature (Pucci, Lynch & Keiper, 2003; Medlock et al., 2018). Thus, in addition to Culicidae, primers were evaluated for non-culicid Nematocera, Hymenoptera, Lepidoptera, Coleoptera and all remaining arthropods.

We ran *ecopcr* with a maximum of five nucleotide differences allowed in either primer and considered the threshold for a primer set’s successful amplification in a given taxonomic group as the presence or absence of the matching 3′ nucleotide in the oligos. The resulting *ecopcr* output was analyzed and graphed in R (R Development Core Team 3.0.1, 2013) using the ROBITools package (http://metabarcoding.org/obitools).

### Mosquito DNA and mock assemblages

Mosquitoes used in pools were collected at various times either in Sao Paulo or Rondônia states, Brazil, and chosen to represent as fully as possible the general Culicidae taxonomic topology. Species used in the mock pools included one individual each of the following taxa: *Aedeomyia squamipennis*, *Aedes* sp., *Anopheles* (*Kerteszia*) sp., *Anopheles* (*Nyssorhynchus*) sp., *Coquillettidia venezuelensis*, *Culex* (*Culex*) sp., *Culex* sp., *Haemagogus* sp., *Limatus* sp., *Mansonia flaveola*, *Mansonia humeralis*, *Mansonia indubitans*, *Mansonia titillans*, *Psorophora* sp., *Wyeomyia* (*Phoniomyia*) sp., and *Wyeomyia* sp. A *Chagasia* sp. DNA sample from the culicid genomic collection at the Faculty of Public Health (University of Sao Paulo) was also used. In all cases, DNA was extracted using a simple salting out protocol preceded by maceration and proteinase K digestion (Sambrook, Fritsch & Maniatis, 2001). Most samples possessed varying degrees of pre- and post-extraction DNA sheering, as verified by agarose electrophoreses (due to sample age or capture method). We therefore did not explicitly measure sample DNA concentration for individual DNA stocks as this would be meaningless in light of degradation. Rather, we utilized the same stocks across the mock pools, thus obviating the need to standardize concentrations.

The DNA from one mosquito was diluted into approximately 300-µL of water and this working stock was used for the creation of four mock genomic DNA assemblies containing only culicid DNA (hereafter *Fresh Pools* A–D). These mixtures were comprised of varying volumes from the 17 working stocks (ranging in contribution from 0.5 to 40 µL of each animal; Data S1). The absolute volume of component taxa varied from 0.28% to 27% of the mock solutions, i.e., two orders of magnitude. The final DNA concentration of the four *Fresh Pools* composite mixtures averaged 0.84-ng/µL. A replicate sequencing run for the *Fresh Pool A* mixture, using different PCR and Illumina sequencing runs, was done to verify the reproducibility of the results (although at a six-fold lower NGS sequencing coverage).

We tested the extent of non-target amplification from the D2 primer pair (described below) by mixing the four *Fresh Pools* with DNA from the combined bycatch of four CDC traps after the maximum care had been taken to remove remnant Culicidae and their tissue, such as loose scales and antennae. The ad hoc identification of CDC content estimated more than 5,000 individuals of varying size (with the bulk of the catch being 5-mm or less) and from the insect orders Blattodea, Coleoptera, Diptera, Hemiptera, Hymenoptera, Lepidoptera, Orthoptera, and several spiders.

Here, the bycatch DNA was extracted as above and used at a concentration of 135 ng/µL in all downstream assays. The CDC bycatch was mixed with of the four *Fresh Pools* in one of two concentrations: 1:1 and 1:10 (1-µL of each *Fresh Pool* to 10-µl of CDC bycatch DNA). Of the resulting eight *Bycatch Pools*, *Bycatch Pool* A1:1 and *Bycatch Pool* A1:10 were submitted to a replicated PCR and sequencing pipeline.

A third set of mock assemblies (named *Degraded Pools* I–IV) was created to test the impact of DNA degradation on the amplification of the D2. These used the same stock genomic DNA solutions for each of the 17 animals as above, after having been left at room temperature for two weeks (an average duration for the field deployment of commonly used attraction traps). Evaporated water was replaced in order to maintain the original concentration. There was also a two orders of magnitude difference between the highest and lowest individual volumes used in each pool (0.5 to 50 µL; Data S1). The degraded DNA mixture averaged 0.74-ng/µL and omitted sample *Wyeomyia* sp.

### PCR parameters

Identical PCRs profiles were used to sequence both the 17 specimens alone and the pooled assemblages. These used Illumina overhang sequences (underlined below) on each primer in a first round PCR:

 Ill+Mozzie.D2.Uni.F (5′-TCGTCGGCAGCGTCAGATGTGTATAAGAGACAGAAGCACTCTGAATAGAGAGTC-3′) andIll+Mozzie.D2.Uni.R (5′-GTCTCGTGGGCTCGGAGATGTGTATAAGAGACAGTGGTCCGTGTTTCAAGAC-3′). The second-round PCR utilized primers designed to include the complements to the Illumina overhangs used in the first PCR, the Illumina sequencing adaptors and Illumina’s 8-bp multiplexing indices.

First-round PCRs were made to 10-µL and included final concentrations of: 1x Phusion HF buffer, 0.2 mM dNTPs, 0.2 µM of each Illumina-overhang primer, 0.2U of Phusion High-Fidelity DNA Polymerase (Thermo Fisher Scientific, USA) and one microliter of template DNA. Hotstart PCRs were initiated at 98 °C using a Eppendorf Mastercycler ep Gradient S followed by the temperature profile: 98 °C (30 sec); 30 cycles of 98 °C (10 sec), 65 °C (30 sec) and 72 °C (30 sec); and a final extension 72 °C for 5 min. Amplicons were visualized on 1% agarose gels and, after confirmation that no primer dimers were present, were diluted 10X prior to the second, indexing PCR. Here, reagent concentrations were as in PCR1, except that the concentration of the indexing primers was 0.075 µM. One microliter of the 10X diluted PCR1 product was used as template. Cycling conditions were: initial denaturation at 98 °C (10 sec); 12 cycles of 98 °C (5 sec), 55 °C (10 sec) and 72 °C (20 sec); and a final extension 72 °C for 1 min.

The products from the second PCR were multiplexed and purified using the QIAquick PCR purification kit (Qiagen, Valencia, CA) and sequenced using MiSeq Reagent v2 Nano chemistry (BPI—Biotecnologia Pesquisa e Inovação, Botucatu, Brazil).

### Sequence processing and statistical analyses

We used MOTHUR v.1.36.1 (Schloss et al., 2009) to create contigs from Illumina Miseq Nano 500 paired-end reads using *deltaq* = 6. Subsequently, the *trim.seqs* command was used to filter sequences with a minimum average quality score of 25 and *maxambig* = 6. Primer sequences were demultiplexed and trimmed using *pcr.seqs* with *pdiffs* = 3, *rdiffs* = 3.

Clustering of reads into zero-radius OTUs (ZOTUs) was done using USEARCH v.11.0.667 with the module *unoise3* (Edgar, 2010), which also removed chimeras based on *de novo* detection. For reads from the sequencing of the 17 un-pooled individuals, ZOTUs represented by fewer than 1% of the total were removed from all subsequent analyses because of the potential of contamination from parts of other species (most specimens were collected *en masse* in CDC traps).

We assigned ZOTUs to taxonomy using the RDP classifier within MOTHUR using *classify.seqs* (Wang et al., 2007) with a D2 database created from all matching GenBank entries and our own sequences derived from the Illumina sequencing of individual animals. The distance matrix used for D2 cladograms was derived using the *calc_distmx* command in USEARCH using default parameters.

We used linear regression to derive R^2^ estimates that could be used to test the predictability of DNA input to read output from Illumina MiSeq sequencing. The regression equation was also used to estimate the slope of the associated regression line and y-intercept. The significance of linear comparisons was assessed using Pearson correlation. The *phytools* R library (Revell, 2012) was used for cladogram estimates and *ggplot2* (Ginestet, 2011) for graph design.

## Results

### Primer efficiency analysis

The initial evaluations for primer design and coverage used all GenBank insect sequences containing at least one of the D2 primers and a 30-bp flank (a total of 5,294 sequences for the forward priming site and 2,582 for the reverse). These included 387 culicid sequences from 233 species and 24 genera for Mozzie.D2.Uni.F. The 146 Mozzie.D2.Uni.R priming sites were form 88 species and 10 genera. Priming sites for the two oligos were found in both Anophelinae and Culicinae.

**Figure 1:**
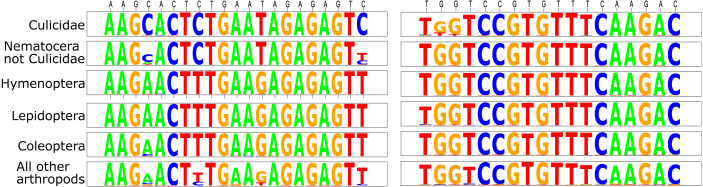
Sequence logos for the D2 primers tested herein. The comparisons are divided among Culicidae, all Nematocera apart from Culicidae, orders that are often collected as bycatch in sampling traps (Hymenoptera, Lepidoptera, and Coleoptera), and the remainder of all arthropods. Primer sequences are defined above the logos in black type (forward primer is listed first).

This initial analysis confirmed that Mozzie.D2.Uni.R was generally conserved across the Culicidae (Fig. 1). Although there were some mismatched base positions in GenBank culicid sequences for this priming site, we believe these were either PCR or sequence-calling errors because of the otherwise high conservation across all Arthropoda (Fig. 1).

In Mozzie.D2.Uni.F, the 3′-cytosine was nearly universal within the Culicidae, but absent in almost all other taxa, thus precluding the polymerase extension of most non-culicids during PCR. Based on the initial search parameters, *Chagasia bathana* was the only culicid entry that did not match the forward primer perfectly; it contained a 3′-thymine rather than cytosine in Mozzie.D2.Uni.F. This potential divergence was addressed herein by the inclusion of a *Chagasia* sp. individual in the pools (see below).

Based on the available GenBank sequences meeting our criteria, the only non-culicid insects with nucleotides identical in both forward (including the 3′-C) and reverse primers belonged to species from closely related Nematocera families.

In the second primer analysis, we used *ecopcr* to evaluate only database entries containing both priming sites. Here, we used a conservative measure of primer amplification efficacy: given that mismatches in the 3′ nucleotide of a primer will theoretically preclude any polymerase extension, and thus PCR, we characterized an oligo’s ability to amplify the markers tested by whether this nucleotide was conserved among the target taxa. The 227 Culicidae sequences meeting the *ecopcr* parameters were from 160 species and 21 genera, including Anophelinae and Culicinae.

As above, there were several cases in Culicidae where either the forward (five sequences) or reverse (60 sequences) primer did not match our D2 templates exactly, we believe these mismatches were likely errors for at least two reasons. First, in all of these cases, there was always another representative of the same species containing the matching template sequence. Moreover, as discussed previously, most mismatched base positions were conserved across several non-culicid taxa (Fig. 1); this was particularly evident for the reverse primer Mozzie.D2.Uni.R, which when submitted to a GenBank BLAST, is fully conserved across nearly all Arthropoda entries, and indeed across many Metazoa (including humans). Second, most of the instances of nucleotide mismatches in the primers occurred near the start of the GenBank entry. This suggests that the discordance was either a Sanger trace miscall or that the primer sequence was not trimmed prior to submission (i.e., the original oligo contained the mismatch). Indeed, our first analysis, which omitted sequences with less than 30 bp flanks, resulted in comparatively fewer mismatched oligo nucleotides.

All GenBank Culicidae D2 sequences used in the *ecopcr* analysis possessed the 3′-C in Mozzie.D2.Uni.F. Diptera taxa containing this nucleotide were almost exclusively Nematocera: Culicidae (17% of the 1,095 sequences with the 3′-C), Simuliidae (7%), Chironomidae (2%), and contributions of less than 5 sequences each from Blephariceridae, Ceratopogonidae, Limoniinae , Lonchopteridae, Micropezidae, Mycetophilidae , Chaoboridae, Dixidae, and Thaumaleidae. There was a sole non-Nematoceran Dipteran sequence from the Drosophilidae (*Drosophila willistoni*, accession number XR_002724221), although another D2 entry from this species did not possess the 3′-C.

Those non-Diptera arthropods possessing the Mozzie.D2.Uni.F 3′-C nucleotide were almost exclusively Amphipoda (Fig. 1). Although many of these taxa occur in saltwater, 63% (689 of the 1,095 sequences containing the 3′-C) included species that can also be found in freshwater: Crangonyctidae, Gammaridae, Haustoriidae, and Niphargidae.

The priming site analysis of the four other metabarcoding primer sets indicated relatively conserved annealing sites in Culicidae for 16S (Figs. S1A and S1B). In CO1, there were comparatively more mismatches due to synonymous third-nucleotide substitutions (Figs. S2A and S2B), including near the 3′-end of the oligo, a potential source of primer bias (Arnheim & Erlich, 1992). In both mitochondrial markers, however, priming sites were more conserved between the various arthropod taxa tested. The 3′ nucleotide position of all primers were generally shared extensively among all arthropods (Figs. S1 and S2).

In terms of taxonomic resolution, the D2 primer set fared well against all other markers previously used for Culicidae metabarcoding (Table 1). Among the “all arthropod” sequence dataset evaluated, only the CO1 locus used by Batovska et al. (2017) had a higher taxonomic resolution (0.910 versus 0.926, respectively). Note that this CO1 dataset contained the commonly used Folmer forward primer LCO1490 (Folmer et al., 1994), so there were relatively few Culicidae GenBank entries because of primer trimming (*n* = 20). Within the Culicidae-only datasets (Table 1), D2 had appreciably better species resolution than the four mitochondrial markers evaluated, including the barcode region of the CO1: 0.969 (D2) versus 0.765 (CO1; Krol et al., 2019) and 0.929 (CO1; Batovska et al., 2017).

**Table 1:** Summary of datasets used to evaluate amplicon length and taxonomic resolution for each primer set discussed in the text. The number of GenBank sequences in the first two columns were those attending the ecopcr maximum nucleotide mismatch of five and that also contained both priming sites.

Marker (reference)	Number of sequences used for all arthropod taxa (mean amplicon length including primers)	Number of sequences used for Culicidae only (mean amplicon length including primers)	Species resolution, all arthropod taxa	Species resolution, Culicidae only
D2 (herein)	15,356 (465 bp)	227 (400 bp)	0.910	0.969
16S (Talaga et al., 2017)	79,039 (205 bp)	83 (217 bp)	0.886	0.838
16S (Schneider et al., 2016)	77,160 (140 bp)	96 (145 bp)	0.867	0.816
CO1 (Krol et al., 2019)	793,484 (154 bp)	13,782 (154 bp)	0.866	0.765
CO1 (Batovska et al., 2017)	3,232 (220 bp)	20 (220 bp)	0.926	0.929

We undertook the same evaluation of taxonomic resolution for only those species that were shared between the D2 and the other datasets in order to address biases due to different taxonomic coverage (Table S1). The primer conservation in this trimmed analysis was similar to that of the complete dataset (see *ecopcr* output files in Data S2). Here too, D2 outperformed the mitochondrial markers in species resolution (Table S1).

A potential explanation for D2’s apparent advantage over previously used metabarcoding primers is that the latter sets were used in *ad hoc* comparisons to differentiate among geographical subsets of mosquitos, rather than the amongst the entire Culicidae (for which the D2 primers were designed). Moreover, the mitochondrial amplicons were less than one half the length of the D2 sequences (Table 1), thus omitting considerable nucleotide variation.

### D2 Illumina sequencing of 17 individual mosquito specimens

An average of 1,244 (SD = 479) Illumina reads were produced across the seventeen individual samples that were subsequently used to create the mock assemblages. We choose this sequencing depth to account for genomic variants in the D2 amplicon, as is common in insects (Tautz et al., 1988).

After primer trimming, the D2 amplicon length for the 17 specimens sequenced was 383 bp (SD = 9) for Culicinae and 432 bp (SD = 24) for Anophelinae. The GC percentage was identical for culicines and anophelines, 60% (SD = 2.5). The only Culicidae GenBank entry with an ambiguous binding site to the primers used herein was *Chagasia bathana* (accession no.: AF417831), which had thymine instead of cytosine in the a 3′-end of Mozzie.D2.Uni.F. The *Chagasia* sp. sample herein yielded successful PCR and sequencing results and there was no indication that it amplified with lower fidelity than other sequences (see below).

The D2 provided unique sequences for each species and also recovered the general relationships of the Culicidae topology (Fig. S3). Most species possessed multiple genomic variants within the same individual, but no sequence was shared between species and the D2 sequences of all animals were reciprocally monophyletic. The variation within individuals was due to both SNPs and indels, the latter being most common in repeating microsatellite regions. There was a total of 59 D2 sequences among the 17 specimens used. These were used to create the D2 library for the taxonomic assignments in the pooled data below. All taxonomic assignments from the Illumina sequencing of pools were based on sequences having 100% identity to those from the sequencing of these individual samples.

### Results for pools

On average, 20% of sequencing output was discarded based on quality filtering parameters (Table S2). We normalized the number of sequence reads (against volume used) in all pools so that comparisons could be made between species and between pools. Following normalization, it is expected that the slope from individual pools would be 1 if all species’ DNA is equally amplifiable. Moreover, the intercept of the regression line is expected to occur near zero if there is a linear relationship between contributed DNA and reads output.

We defined a Best Estimate of the true contribution of each stock DNA by averaging the normalized read outputs for each of the 17 species from the four *Fresh Pools* (A–D) (Table S3). This is in effect an estimate of what percentage of reads would results if pools were created from equal initial volumes of the 17 stock solutions. The least amplicon-dense genomic DNAs were generally from older specimens or animals that had been collected in CDC traps exposed to rain.

Pearson correlation was used to test significant association between both axes in the regression calculations. There was a highly significant correlation for all comparisons (<0.01), even after adopting Bonferroni corrections for multiple tests, except in comparisons involving the 1:10 dilutions with bycatch, for which no significance was found in any of the pools (but see below).

### Culicid-only pools

Table S3 and Fig. S4 present the normalized proportion of sequence reads resulting from the stock DNA averaged across the four *Fresh Pools* and across the four *Degraded Pools*. In both mixture types, the individual possessing the highest proportion of amplifiable targets was *Mansonia flaveola* (this specimen had been captured and its DNA preserved immediately in the field, unlike most other specimens). Although the taxon with the lowest proportion of amplified D2 in the *Fresh Pools* was *Psorophora* sp., *Chagasia* sp. was by far the least amplicon-dense taxon in the *Degraded Pools*. This, and other similar disparities vis-à-vis sample degradation may be due to differences in DNA elution solutions leading to higher degradation at room temperature.

A strong linear relationship exists in the standardized values between fresh and degraded pools (R^2^ = 0.94, slope=1.05, *y*-intercept = −0.004; Fig. S4). Note however, that because of space constraints on the flow cell, the coverage for *Degraded Pools* was, on average, five times less than those from the *Fresh Pools* (Table S2 and Data S1). Thus, there is likely some variation due to sample size effects, in addition to DNA degradation.

In the five *Fresh Pools*, all 17 taxa were consistently recovered, including for the low-coverage replicate of *Fresh Pool* A (A-REP in Table S2). In these mixtures, the RDP taxonomic assignments recovered Diptera sequences in 99% of cases, the remaining balance consisted of PCR artifacts, generally of very short sequences.

**Figure 2:**
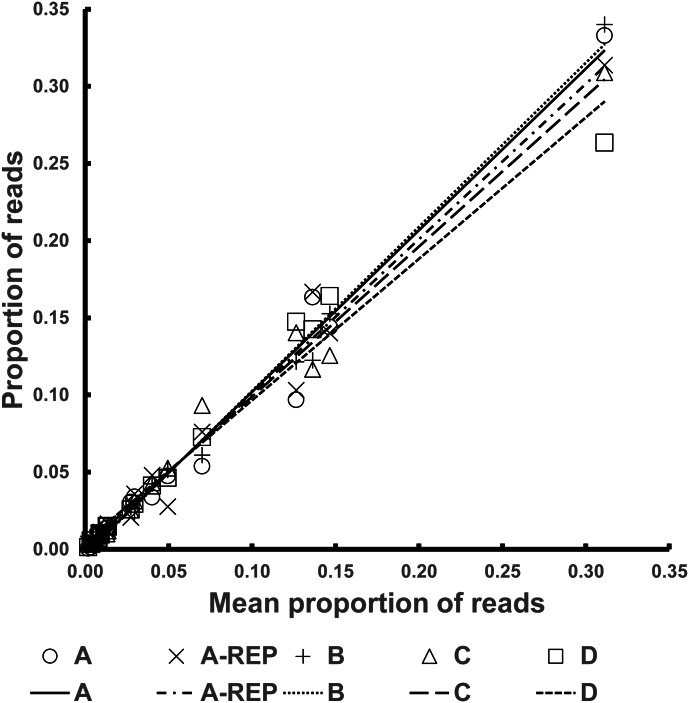
Representation of the normalized read counts extrapolated from the average of the four *Fresh Pools* (A–D) for each species (*x*-axis) and the values from the four individual pools (*y*-axis).

The R^2^ values of the *Fresh Pools* were all above 0.97. Graphical representation of the Best Estimate values against those for the four individual pools can be seen in Fig. 2.

Within the *Degraded Pools*, several species sometimes failed to be recovered: *Chagasia* sp., *Culex* (*Culex*) sp., *Psorophora* sp., and *Mansonia humeralis* (Fig. S5 and Data S1). This was not unexpected, as in all these cases of species dropout, the proportion of degraded DNA added was 2% or less of the pool volume (Data S1). Moreover, the sequencing depth for the *Degraded Pools* was on average five times lower than that for the *Fresh Pools.*

### Bycatch pools

All reads from *Bycatch Pools* were assigned to the order Diptera (RDP), highlighting the low cross-reactivity of the designed primers. Here, comparatively fewer Culicidae reads resulted, as the primers amplified an additional five Diptera families (Table S2). By far, the main bycatch taxon represented was Chaoboridae, which has been proposed as the most phylogenetically related family to Culicidae (Kumar & Rai, 1990). The replicate samples for both the 1:1 and 1:10 mixtures of *Bycatch Pool* A tended to yield similar proportions of non-target reads.

Although the utmost care was taken to remove all remnant culicid tissue from the CDC bycatch prior to creating the *Bycatch Pools*, we believe that carryover nonetheless impacted the 1:1 and 1:10 mixtures, as it comprised 160 and 1,600 times more DNA (respectively) than the mosquitos in these pools. This is supported by the fact the more bycatch-biased pools (1:10) do not have 10 times fewer culicid reads, as would be expected (Table S2). We thus excluded from the regression analyses all specimens that had been captured sympatrically with the CDC bycatch (see Table S3).

The only 1:1 *Bycatch Pool* where a species was not recovered in the sequence reads was *Bycatch Pool* D, which could not recover the *Anopheles* (*Kerteszia*) sp. contribution. This may be a result of: low sequencing coverage (this pool yielded only 6,358 reads), low amplification density per stock DNA volume (Table S3) and because the *Anopheles* (*Kerteszia* sp.) contributed to this pool only 0.3 % of the final volume (Data S1).

**Figure 3:**
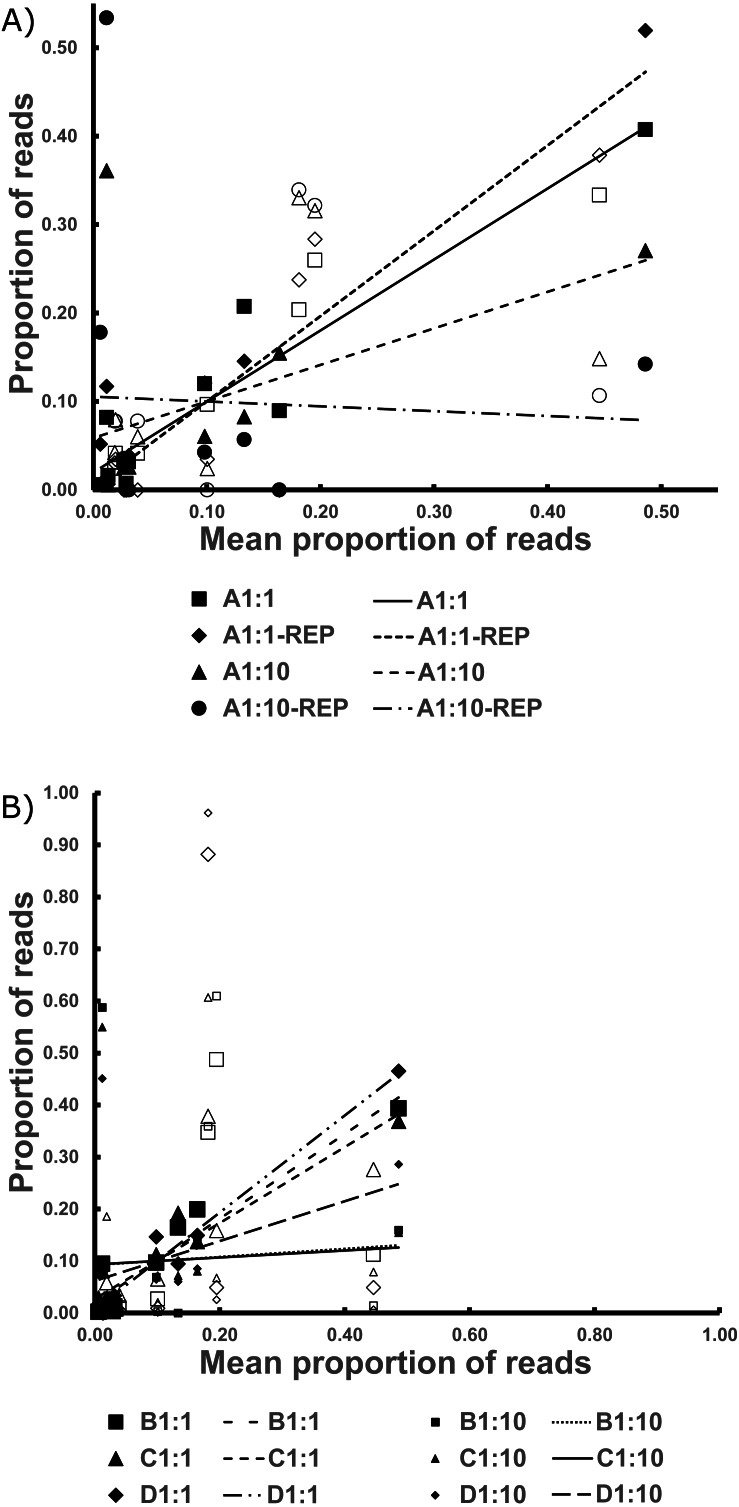
The Best Estimate values (*x*-axis) against the estimates from standardized values in the Bycatch Pools (*y*-axis). (A) The 1:1 and 1:10 A Bycatch Pools and their replicates. (B) The two dilution types for Bycatch Pools B–D. Empty icons were from “contaminant” specimen tissue within the CDC DNA, as discussed in the text. Regression lines were drawn based only on the non-contaminant specimens.

The replicates for *Bycatch Pools* A 1:1 and A 1:10 (suffixed with “-REP” in Table S2) underwent separate PCRs and were sequenced on a different Illumina run from the original. Both replicates failed to recover any sequences for 4 of the 17 DNA samples PCRed. However, these had, on average, six times lower coverage than the original iterations (average of 2,728 reads per replicate; Table S2). The coefficient of determination of the 1:1 dilution was over 0.9. However, the R^2^ values were very low in the 1:10 dilution because of species drop-out and the artificial enrichment for the contaminant sequences discussed above (Fig. 3, Table 2 and Data S1).

## Discussion

Culicidae monitoring schemes are a critical pillar of the integrated control of nuisance and vector species (Gubler, 2002). Diagnosing the taxa present or absent in populated areas enables the timely control of disease vectors (Weaver, 2013). Monitoring non-vector biodiversity is equally relevant because interspecific interactions can modulate the epidemiological dynamics of several vector-borne pathogens. For example, some ornithophilic species of *Culex*, which rarely bite humans, are nonetheless important in the cycling of West Nile Virus in avian reservoirs (Sardelis et al., 2001; Kilpatrick et al., 2005; Cruz-Pacheco, Esteva & Vargas, 2012).

It is thus important to establish a quantitative framework that can track the entire mosquito biodiversity and abundance through time and space, not merely vector species’ presence or absence. Moreover, given the extensive phylogenetic distribution of vector and nuisance taxa, nearly all culicid genera must be monitored with equal efficiency. However, there is as of yet no standardized metabarcoding protocol for such (Chaves et al., 2016; Batovska et al., 2018).

Mosquitoes are particularly amenable to high throughput metabarcoding because most taxa fall within a narrow biomass range. Thus, extremely large species within the family will not contribute disproportionate amounts of DNA and, consequently, skew biodiversity results by causing the drop-out of rarer taxa. Moreover, monitoring efforts now benefit from several iterations of attractant traps (baited with visual, olfactory and temperature cues) that can be deployed for up to several weeks and allow intensive spatial and temporal sampling (e.g., Rubio-Palis et al., 2012).

Here we present a protocol using a universal Culicidae primer set that is effective with both baited traps targeting hematophagous insects or can be used with sampling regimes that capture larger proportions of non-target taxa.

Most metabarcoding protocols have used the popular CO1 mitochondrial locus because it can diagnose inter- (and often intra-) specific differences and because it occurs in multiple copies, and thus is appropriate for minute or degraded samples (Hebert & Gregory, 2005). However, CO1 has limitations that may not favor its use in mosquito monitoring efforts. First, there exist, as of yet, no fully conserved primers that target across Culicidae without taxon-specific amplification bias. Consequently, most CO1 work has made use of nucleotide degeneracy at several priming sites, particularly in the reverse primer (Batovska et al., 2018).

**Table 2:** Regression line parameters for the pools created from the bycatch mixed DNA (created from either 1:1 or 1:10 mixtures of fresh pools A–D with CDC bycatch) and degraded DNA (I–IV). The results for the bycatch mixtures and the first four columns of pools I–IV are regressed against the standardized averages of the four pure-culicid *fresh pools*. The second I–IV regression parameters are based on a regression against the average of the standardized output for each species from those same pools.

	A	A-REP	B	C	D	A1:1	A1:1-REP	A1:10	A1:10-REP	B1:1	B1:10	C1:1	C1:10	D1:1	D1:10	I	II	III	IV
R^2^	0.981	0.980	0.993	0.985	0.974	0.888	0.807	0.237	0.002	0.914	0.004	0.911	0.004	0.947	0.141	0.942	0.856	0.961	0.898
slope	0.937	0.973	0.934	1.012	1.063	1.413	1.004	0.916	−0.061	3.548	0.668	1.962	0.177	6.441	10.545	1.057	0.964	1.047	1.029
y-intercept	0.004	0.002	0.004	−0.001	−0.004	−0.003	0.005	0.013	0.031	−0.004	0.029	−0.007	0.029	0.000	0.019	−0.004	0.001	−0.003	−0.002

In light of these limitations, mosquito CO1 metabarcoding, when attempted, has been tailored to amplify only a subset of Culicidae species, usually those thought to inhabit a given study region (e.g., Batovska et al., 2018). This considerably limits applicability vi-a-vis exotic species detection and scenarios where broad detectability is needed.

Conversely, the 16S mitochondrial marker has been used as a fully conserved primer, but it also efficiently amplifies many other insect groups (Schneider et al., 2016), thus leading to data loss because of flow cell saturation from non-target amplicons. Results herein confirm this drawback for primer sets previously used to amplify these mitochondrial markers (Figs. S1A and S1B).

Unlike protein-coding markers (such as CO1), D2 is less susceptible to nucleotide mutational saturation, particularly at third codon coding positions (Gillespie et al., 2005). This has particular implications for the design of tailored primers that exclude non-target taxa. In rDNA there is an approximate linear relationship between taxonomic distance and nucleotide divergence at priming sites; unlike coding markers that characteristically plateau due to saturated base positions (Deagle et al., 2014). Because of this linearity, nucleotides can often be incorporated, especially in the oligos’ 3′-ends, that allows for amplification of only a chosen taxonomic level (Zhang & Hewitt, 1997), as was done herein (Fig. 1).

The very high coefficients of determination for both the pure *Fresh Pools* (A–D) and the 1:1 *Bycatch Pool* dilutions fare well against previously published studies. For example, the *Fresh Pools* are as or better at predicting sequence reads from initial DNA volume than both the 28S D6 region and mitochondrial CO1 used by (Krehenwinkel et al., 2017). The results from mixed samples, which were confounded by both bycatch noise and potential culicid trace contamination (described above), generally produced accurate biodiversity estimates in the 1:1 dilutions (that is, for *Fresh Pools* A–D diluted 1:1 with bycatch DNA). However, the mixtures with hyper-saturations of bycatch (i.e., 1:10 pools, containing 1,600 more bycatch than culicid DNA) were less correlated to the expected values due to species dropout from low sample size. (It should be noted that the 1:1600 assemblage is probably an exaggerated ratio of the mosquito to non-mosquito biomass normally caught in most currently used surveillance traps).

In insects, the expansion segments of the 28S rRNA operon are highly variable and can be used for interspecific discrimination in mosquitoes (Krzywinski, Wilkerson & Besansky, 2001; Sallum et al., 2002; Beebe, 2018). Although structural mutations tend to be balanced on stem regions by compensatory changes, the relative functional elasticity of these segments within the completed ribosome (especially in loop regions) leads to substantial nucleotide variability (Gillespie et al., 2005). Here, we highlight that the D2 amplicon used outperforms mitochondrial markers CO1 and 16S in its species resolution capacity for Culicidae (Table 1 & Table S1).

There is little empirical evidence comparing the diagnostic performance of D2 against ITS2, the most used mosquito ribosomal DNA locus. Both markers have been historically overlooked, at least partly, because of rDNA’s propensity for length variation, which complicated analyses of Sanger sequence traces (Sallum et al., 2002; Bower, Cooper & Beebe, 2009). Even when intragenomic variation is low (i.e., there are few enough variants that Sanger traces can extrapolate each), the variation in peak height is not always representative of the actual contribution from the component amplicons (Thomann et al., 2002). Although the highly polymorphic ITS2 is an appropriate candidate for culicid monitoring, we failed to find primers for this locus that were universally conserved within Culicidae and that did not amplify non-culicids (the two criteria needed for such metabarcoding).

Importantly in the context of accurate species diagnosis using D2 and other rDNA markers, is that concerted evolution tends to homogenize variants within panmictic populations and, by extension, species. This process may often be more effective for species delimitations than other markers that suffer from protracted mutation/drift lineage sorting or introgression of mitochondrial genes (Hillis & Dixon, 1991). In Culicidae, this characteristic results in intraspecific rDNA variation tending to be lower and more monophyletic. For example, whereas paraphyletic CO1 markers are not congruent with biological species limits, ribosomal DNA can often recover the correct assignments (Ambrose et al., 2012; Beebe, 2018).

The relatively short D2 amplicon is also an important consideration in the context of NGS and metabarcoding, which currently is limited to less than ∼600 bp on the Illumina MiSeq platform. Although there was no apparent bias in our results, the differing amplicon lengths between Culicinae (383 bp, SD = 9) and Anophelinae (432 bp, SD = 24) should be considered in the design and data analysis of future experiments because shorter variants tend to PCR-amplify in higher proportion in mixed-length templates.

Finally, although polymerase elongation may be attenuated in GC-rich regions, such as are common in rDNA (McDowell, Burns & Parkes, 1998), we found little variation in GC-content across the Culicidae D2 (60% in both subfamilies).

### Important considerations

The adoption of modern traps in monitoring efforts represents an unprecedented ability to sample *en masse* and efficiently evaluate an area’s culicid biodiversity. The molecular protocol designed herein can benefit monitoring programs that utilize traps aimed specifically at hematophagous insects (e.g., Mosquito Magnet®) and can collect tens of thousands of culicids. Moreover, the primers’ amplification fidelity across primarily Culicidae also allows processing of field collections with substantial amounts of bycatch (e.g., CDC or Malaise traps, Govella et al., 2011). Notably, some of the non-culicid bycatch detected in the Illumina sequences was nonetheless also epidemiologically relevant, such as Ceratopogonidae, which comprises biting midges responsible for transmitting several human and animal diseases (Table S2). Additional work may elucidate if the D2 primer set can also be applicable in the monitoring of these dipterans.

Estimating the required sequencing depth for metabarcoding is an important consideration in terms of both time and resource efficiencies. In the *Fresh Pools* (which contained only Culicid DNA), each of the 17 species were consistently recovered in both pools A–D (averaging 17,000 reads per Illumina run) and the replicate pool A-REP (3,000 reads). Given the original contribution of stock DNA volume to these pools was as low as 0.2% (Data S1), within a field-collected sample (everything else being equal), the likelihood of identifying a single mosquito from a culicid-only pool of 500 is reasonable even at sequence coverage of less than 5,000 reads/pool. The MiSeq Reagent Kit v2 Nano reaction used herein yields up to one million paired-end sequences for less than US$1000, potentially allowing for multiplex sequencing of hundreds of bulk pools containing hundreds of thousands of mosquitos. In sampling scenarios with substantial bycatch, there is a need to (at least crudely) estimate the relative concentration of bycatch in order to establish the sequencing depth needed to identify all mosquito species in a collection.

Many sampling apparatuses used in culicid monitoring can be deployed during several weeks and lead to DNA and tissue loss. Here, we show that pools simulating this degradation during trap deployment will not significantly bias abundance estimates, likely due to the size of the target amplicon. Moreover, we show that even if these traps capture a substantial proportion of non-culicids, the original mosquito biodiversity will still be recoverable (with enough sequencing coverage).

Given the culicid life cycle, the primers described herein also offer substantial advantages for detection of environmental DNA (eDNA) from immatures in aquatic systems (Schneider et al., 2016). Although eDNA produces unprecedented sensitivity to mosquito presence/absence and substantially lowers sampling effort, the protocol also captures an exponentially higher ratio of bycatch DNA, given the relatively scarce aquatic culicid eDNA (Boerlijst et al., 2019). In such a context, culicid-specific primers would at worse decrease the sampling depth needed for monitoring and at best lead to the diagnosis of only the mosquito eDNA. Here, a potential drawback of the D2 primers is their co-amplification of amphipods, particularly freshwater Gammarus species (“All other arthropods” in Fig. 1). Of the GenBank sequences that possessed the 3′-C of the forward primer in the *ecopcr* analysis (*n* = 1095), 65% belonged to the amphipods (these represented the only instances where this nucleotide was present in non-Dipteran arthropods). Although this is not an issue in many scenarios, such as monitoring of mosquitos that breed in containers or temporary pools, *ad hoc* consideration should be given to data contamination from these animals, particularly when working with environmental DNA. Of course, this is not an issue with adult captures.

Another important caveat in ascertaining the reliability of a rDNA marker for abundance estimates is the potential for sexually dimorphic genomic D2 copies in Anophelinae. In this subfamily, the location and size of the nucleolus organizer regions (NORs) are phylogenetically inconsistent and D2 amplification may vary between homo- and heteromorphic chromosomes (Rafael, Tadei & Recco-Pimentel, 2003; Wilkins, Howell & Benedict, 2007). In Culicinae, an equal contribution of rDNA amplicons is expected, as here ribosomal DNA is concentrated in homomorphic chromosomes (Kumar & Rai, 1990).

The primer-design strategy described herein is likely adaptable to non-Culicid Metazoa taxa given the abundance of rDNA sequences on public databases. Primer design can be facilitated through available bioinformatics tools (e.g., Riaz et al., 2011; Boyer et al., 2016).

##  Supplemental Information

10.7717/peerj.9057/supp-1 Data S1 Dilutions used and Illumina read outputs for each pool Absolute and proportional volumes of genomic DNA used per specimen are listed in the tabs ”dillutions Fresh Pools A-D” and ”dillutions Degraded Pools I-IV”. Resulting absolute and proportional Illumina read outputs are listed in tabs ”Fresh Pool (A-D) reads #” and ”Degraded Pools (I-IV) reads #”.

10.7717/peerj.9057/supp-2 Data S2 EcoPCR results for comparisons using D2 and 16S/CO1 Ecopcr results for comparisons between D2 and 16S and D2 and CO1. The parameters are described for only those species that are shared between amplicons of the respective comparisons.

10.7717/peerj.9057/supp-3 Figure S1 Sequence logos for mitochondrial DNA marker 16S Sequence logos of primers for two 16S amplicons that have previously been used for culicid metabarcoding. Primer sequences are defined above the logos in black type (forward primer is listed first). Primers amplified a target averaging 210 bp for A) and 142 bp for (B).

10.7717/peerj.9057/supp-4 Figure S2 Sequence logos for mitochondrial DNA marker CO1 Sequence logos of primers for two CO1 amplicons that have previously been used for culicid metabarcoding. Primer sequences are defined above the logos in black type (forward primer is listed first). Primers amplified an average of 154 bp for (A) and 220 bp for (B).

10.7717/peerj.9057/supp-5 Figure S3 Neighbor joining cladogram of D2 sequences Neighbor joining cladogram of D2 sequences from each of the 17 specimens used to create the mock DNA pools. Numbers in parentheses indicate the proportion of that variant within each animal.

10.7717/peerj.9057/supp-6 Figure S4 Regression line for the average read contribution from the seventeen species analyzed in culicid-only mixes Regression line for the average read contribution from the seventeen species analyzed in culicid-only mixes:*Fresh Pools* (A-D) (x-axis),*Degraded Pools* (I-IV) (y-axis)

10.7717/peerj.9057/supp-7 Figure S5 Standardized read quantity for each of the 16 species used in*Degraded Pools* Standardized read quantity for each of the 16 species used in*Degraded Pools* I-IV (y-axis) against the Best Estimate from the four*Fresh Pools* (A-D) (x-axis).

10.7717/peerj.9057/supp-8 Table S1 Evaluation of the taxonomic resolution between the D2 primer set designed herein and other primers tested when only shared Culicidae species are considered Evaluation of the taxonomic resolution between the D2 primer set designed herein and other primers tested when only shared Culicidae species are considered (there were no shared taxa between D2 and Batovska*et al*., 2017). The last two columns list the number of D2 sequences used in the respective comparisons and the taxonomic resolution calculated from these.

10.7717/peerj.9057/supp-9 Table S2 Sequencing output for each of the pools used and the taxonomic assignments of the quality-filtered reads Sequencing output for each of the pools used and the taxonomic assignments of the quality-filtered reads. The three iterations of the A pool were done in replicate (denoted by “-REP”).

10.7717/peerj.9057/supp-10 Table S3 Estimated proportions of the reads each animal contributed to a pool after standardization by volume Estimated proportions of the reads each animal contributed to a pool after standardization by volume. Column A is the mean of the four standardized*Fresh Pools* (A-D). Column B is the average of the standardized*Degraded Pools* (I-IV). Asterisks indicate individuals that were removed from the same CDC samples used in the*Bycatch Pools* (collected in Rondônia State).

## Additional Information and Declarations

### Data Availability

The following information was supplied regarding data availability: The DNA dilutions used and Illumina read outputs for each pool are available in Data S1. EcoPCR results for comparisons using D2 and 16S/CO1 are available in Data S2. The sequences are available in the NCBI Sequence Read Archive: PRJNA575712.
