# Supplementary material for: Culicidae-centric metabarcoding through targeted use of D2 ribosomal DNA primers
Source: PeerJ. 2020 Jun 3;8:e9057. doi: 10.7717/peerj.9057 (PMC7315618; doi:10.7717/peerj.9057)
Supplement: Figure S1 — Sequence logos of primers for two 16S amplicons that have previously been used for culicid metabarcoding. Primer sequences are defined above the logos in black type (forward primer is listed first). Primers amplified a target averaging 210 bp for A) and 142 bp for (B). [file peerj-08-9057-s003.pdf]

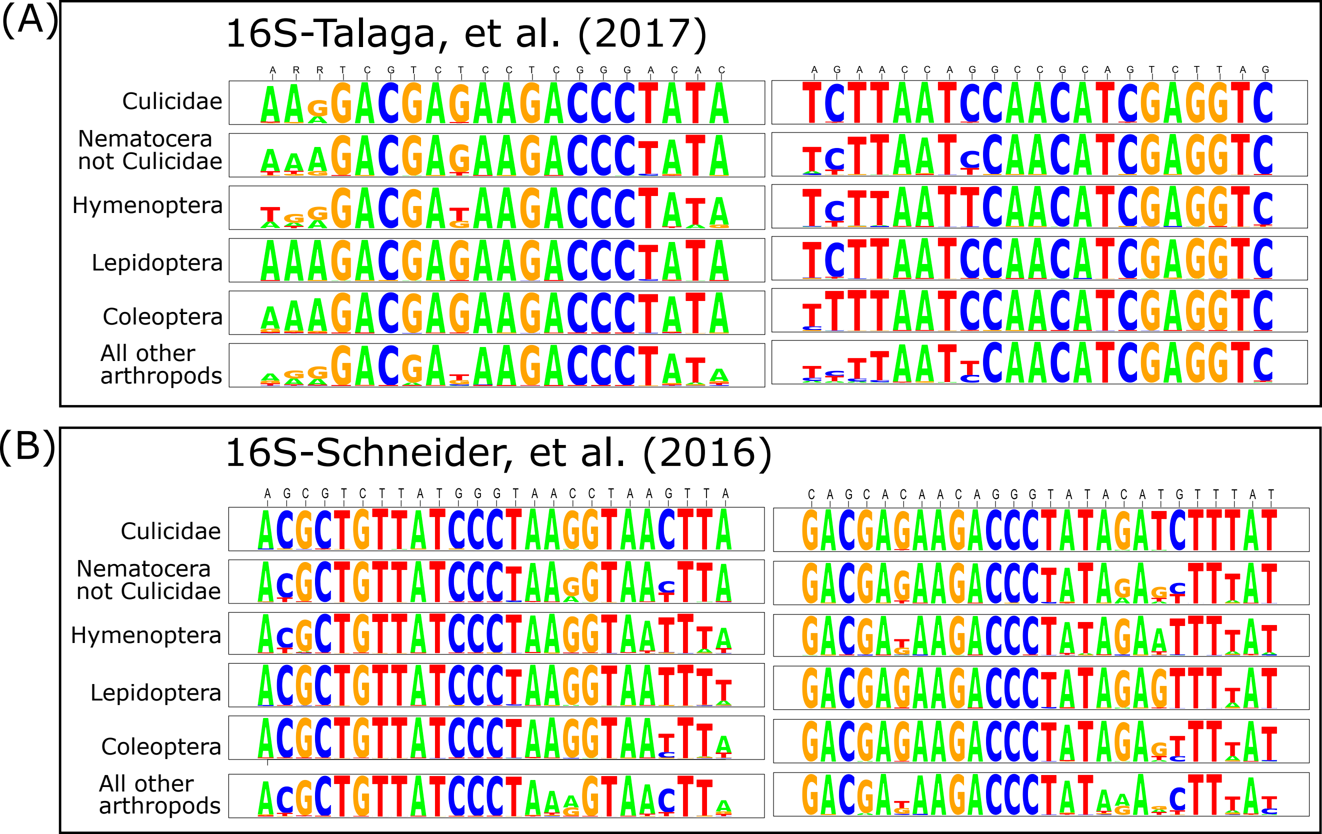

(A)
16S-Talaga, et al. (2017)
Culicidae
Nematocera not Culicidae
Hymenoptera
Lepidoptera
Coleoptera
All other arthropods
(B)
16S-Schneider, et al. (2016)
Culicidae
Nematocera not Culicidae
Hymenoptera
Lepidoptera
Coleoptera
All other arthropods
