# Supplementary figures and images for: Culicidae-centric metabarcoding through targeted use of D2 ribosomal DNA primers
Source: PeerJ. 2020 Jun 3;8:e9057. doi: 10.7717/peerj.9057 (PMC7315618; doi:10.7717/peerj.9057)

(A)

## CO1-Krol, et al. (2019)

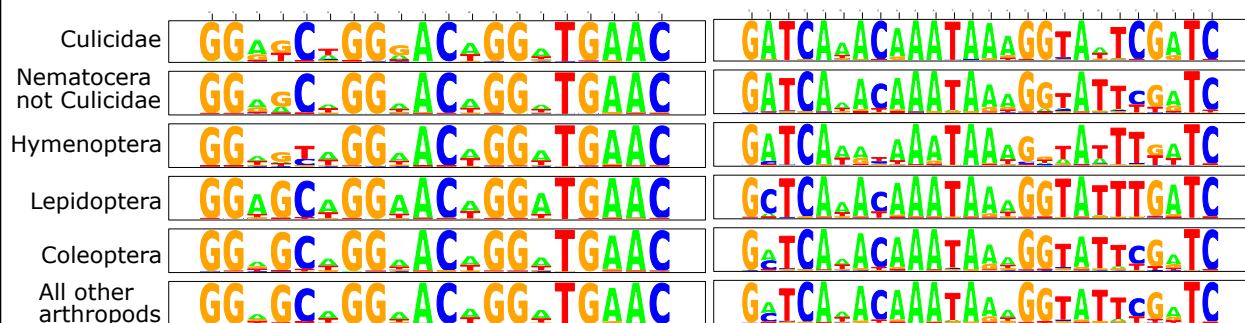

(B)

## CO1-Batovska et al., 2017

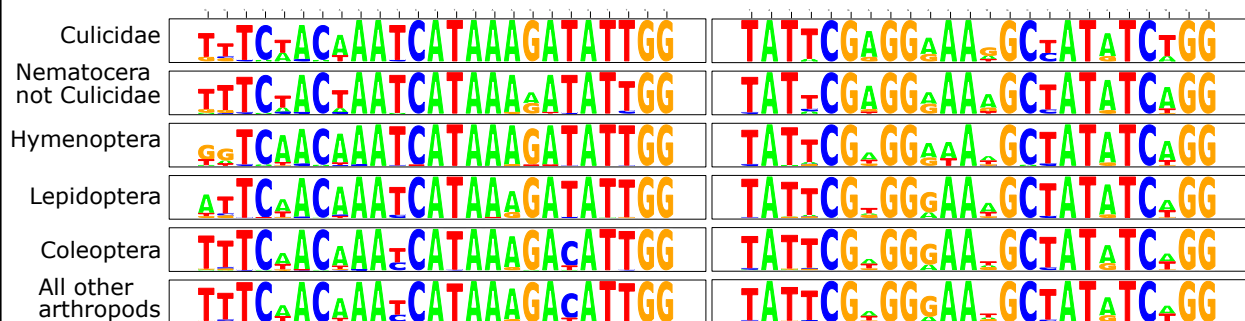

Supplement: Figure S2 — Sequence logos of primers for two CO1 amplicons that have previously been used for culicid metabarcoding. Primer sequences are defined above the logos in black type (forward primer is listed first). Primers amplified an average of 154 bp for (A) and 220 bp for (B). [file peerj-08-9057-s004.pdf]

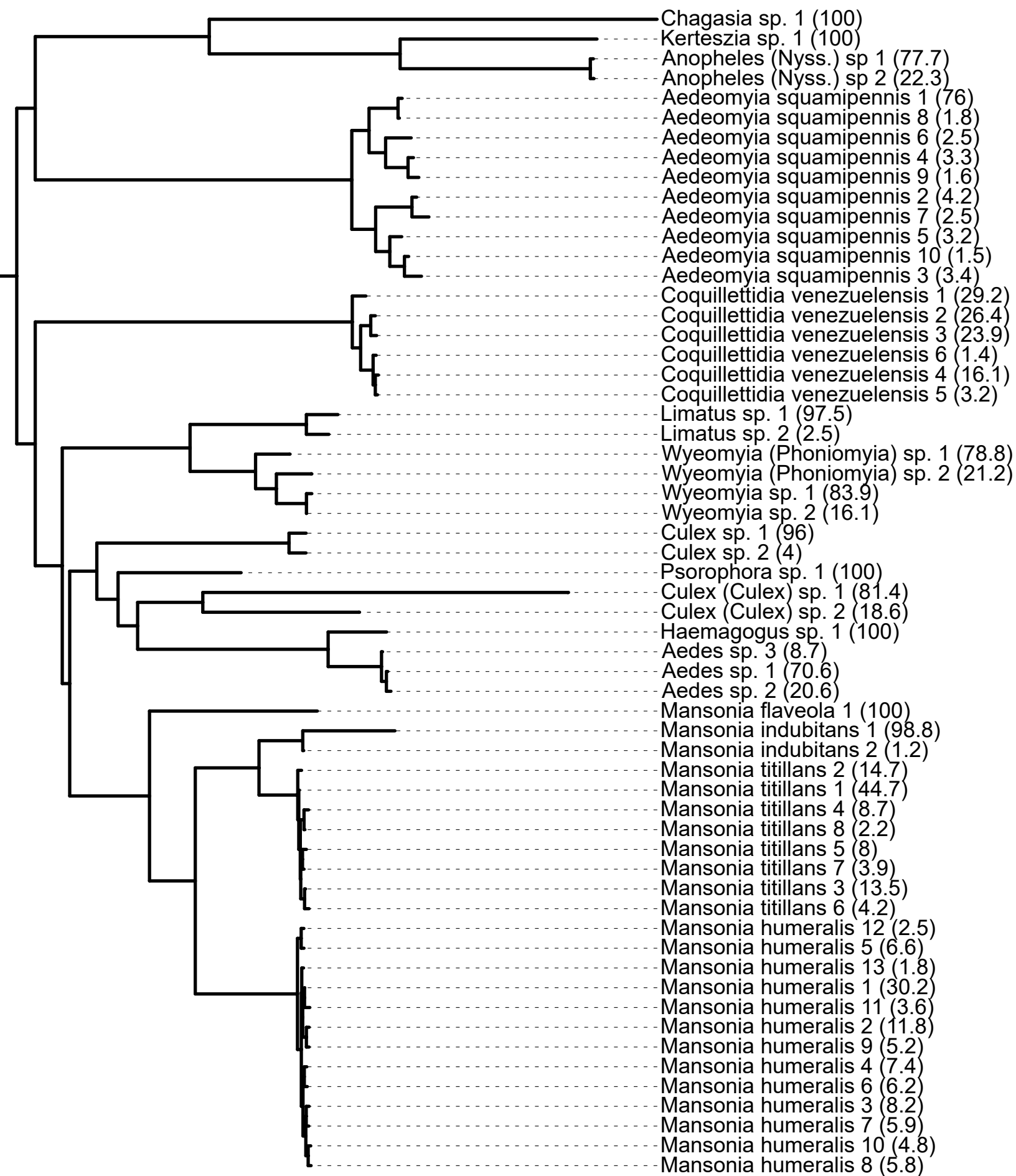

Supplement: Figure S3 — Neighbor joining cladogram of D2 sequences from each of the 17 specimens used to create the mock DNA pools. Numbers in parentheses indicate the proportion of that variant within each animal. [file peerj-08-9057-s005.pdf]

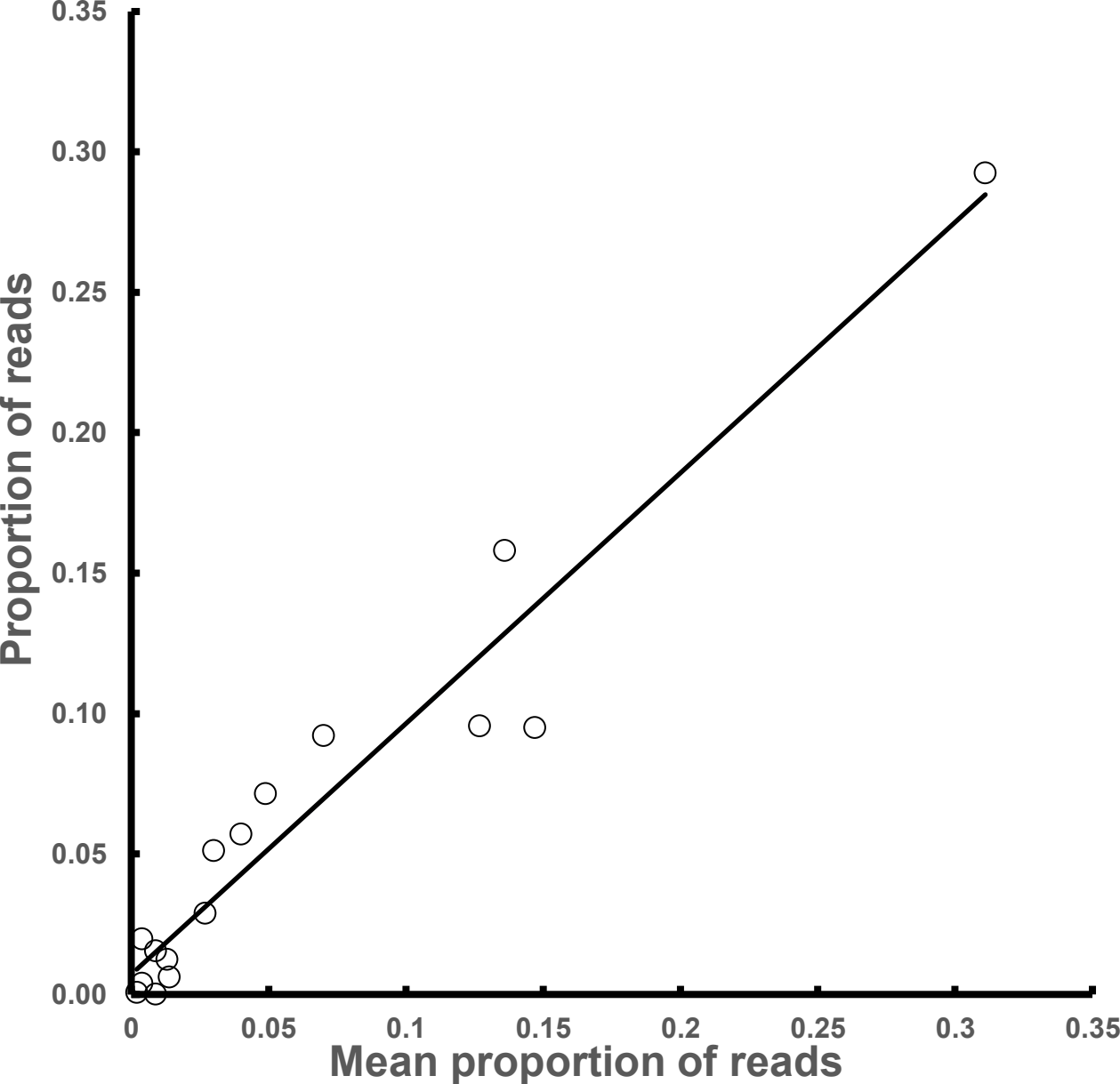

Supplement: Figure S4 — Regression line for the average read contribution from the seventeen species analyzed in culicid-only mixes:Fresh Pools (A-D) (x-axis),Degraded Pools (I-IV) (y-axis) [file peerj-08-9057-s006.pdf]

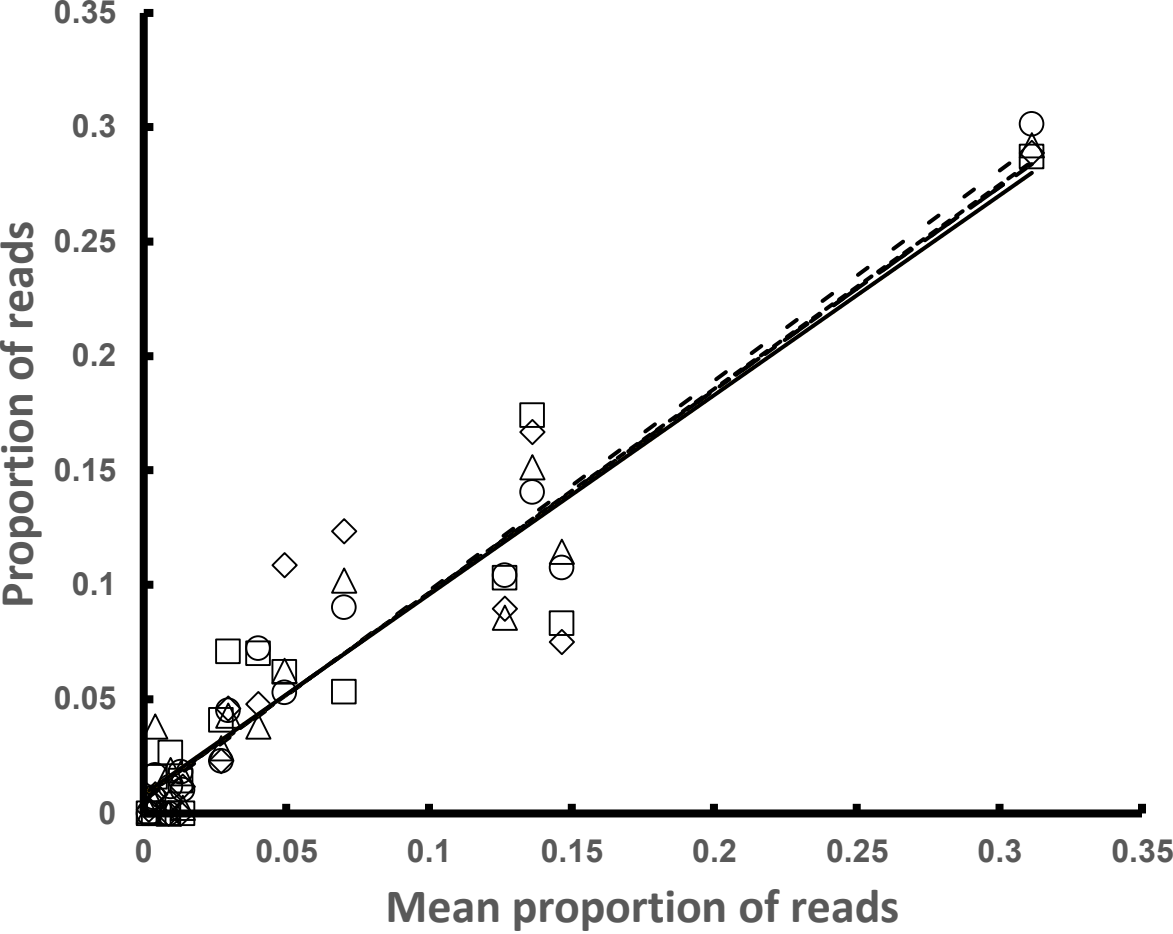

△ I    ◇ II    ○ III    □ IV    ..... I    --- II    - - III    — IV

Supplement: Figure S5 — Standardized read quantity for each of the 16 species used inDegraded Pools I-IV (y-axis) against the Best Estimate from the fourFresh Pools (A-D) (x-axis). [file peerj-08-9057-s007.pdf]
