# Supplementary material for: Culicidae-centric metabarcoding through targeted use of D2 ribosomal DNA primers
Source: PeerJ. 2020 Jun 3;8:e9057. doi: 10.7717/peerj.9057 (PMC7315618; doi:10.7717/peerj.9057)
Supplement: Table S1 — Evaluation of the taxonomic resolution between the D2 primer set designed herein and other primers tested when only shared Culicidae species are considered (there were no shared taxa between D2 and Batovskaet al., 2017). The last two columns list the number of D2 sequences used in the respective comparisons and the taxonomic resolution calculated from these. [file peerj-08-9057-s008.docx]

| Marker (reference) | Unique species available that are shared with D2 (herein) | Number of Culicidae sequences used in the comparison with D2 | Culicidae species resolution | Number of Culicidae D2 sequences used in comparison | Species resolution for D2 |
| --- | --- | --- | --- | --- | --- |
| 16S (Talaga, *et al*., 2017) | 15 | 57 | 0.882 | 41 | 1.000 |
| 16S (Schneider, *et al*., 2016) | 15 | 66 | 0.882 | 41 | 1.000 |
| CO1 (Krol, *et al*., 2019) | 92 | 7,613 | 0.809 | 146 | 0.957 |
