# Supplementary material for: Culicidae-centric metabarcoding through targeted use of D2 ribosomal DNA primers
Source: PeerJ. 2020 Jun 3;8:e9057. doi: 10.7717/peerj.9057 (PMC7315618; doi:10.7717/peerj.9057)
Supplement: Table S2 — Sequencing output for each of the pools used and the taxonomic assignments of the quality-filtered reads. The three iterations of the A pool were done in replicate (denoted by “-REP”). [file peerj-08-9057-s009.docx]

|  |  | Total number of post-filtering reads (% of total) | Culicidae (%) | Ceratopogonidae (%) | Chaoboridae (%) | Chironomidae (%) | Perissommatidae (%) | Sciaridae (%) |
| --- | --- | --- | --- | --- | --- | --- | --- | --- |
| Fresh pools | A | **19194 (80)** | **99** | **0.0** | **0.0** | **0.0** | **0.0** | **0.0** |
|  | A_REP | **3035 (78)** | **99** | **0.0** | **0.0** | **0.0** | **0.0** | **0.0** |
|  | B | **20855 (83)** | **99** | **0.0** | **0.0** | **0.0** | **0.0** | **0.0** |
|  | C | **15642 (83)** | **99** | **0.0** | **0.0** | **0.0** | **0.0** | **0.0** |
|  | D | **13676 (81)** | **99** | **0.0** | **0.0** | **0.0** | **0.0** | **0.0** |
| Mixed pools (1:1) | A_1:1 | **18528 (81)** | **43** | **3.3** | **44.6** | **9.0** | **0.0** | **0.1** |
|  | A_1:1_REP | **2536 (85)** | **47** | **1.9** | **43.2** | **7.8** | **0.2** | **0.0** |
|  | B_1:1 | **19915 (87)** | **43** | **3.3** | **44.5** | **9.4** | **0.1** | **0.1** |
|  | C_1:1 | **20193 (85)** | **48** | **2.9** | **40.9** | **8.2** | **0.0** | **0.1** |
|  | D_1:1 | **22076 (86)** | **36** | **3.4** | **49.3** | **11.2** | **0.1** | **0.1** |
| Mixed pools (1:10) | A_1:10 | **17059 (87)** | **28** | **4.4** | **54.5** | **13.3** | **0.1** | **0.1** |
|  | A_1:10_REP | **2614 (86)** | **28** | **3.5** | **58.4** | **9.8** | **0.1** | **0.0** |
|  | B_1:10 | **19361 (88)** | **27** | **4.2** | **55.9** | **13.2** | **0.1** | **0.1** |
|  | C_1:10 | **20045 (86)** | **27** | **4.1** | **57.0** | **12.2** | **0.1** | **0.1** |
|  | D_1:10 | **24165 (85)** | **26** | **4.6** | **56.1** | **13.4** | **0.1** | **0.1** |
| Degraded pools | Pool_I | **4219 (79)** | **100** | **0.0** | **0.0** | **0.0** | **0.0** | **0.0** |
|  | Pool_II | **5233 (88)** | **100** | **0.0** | **0.0** | **0.0** | **0.0** | **0.0** |
|  | Pool_III | **3118 (70)** | **100** | **0.0** | **0.0** | **0.0** | **0.0** | **0.0** |
|  | Pool_IV | **3260 (78)** | **100** | **0.0** | **0.0** | **0.0** | **0.0** | **0.0** |
