# Supplementary material for: Culicidae-centric metabarcoding through targeted use of D2 ribosomal DNA primers
Source: PeerJ. 2020 Jun 3;8:e9057. doi: 10.7717/peerj.9057 (PMC7315618; doi:10.7717/peerj.9057)
Supplement: Table S3 — Estimated proportions of the reads each animal contributed to a pool after standardization by volume. Column A is the mean of the four standardizedFresh Pools (A-D). Column B is the average of the standardizedDegraded Pools (I-IV). Asterisks indicate individuals that were removed from the same CDC samples used in theBycatch Pools (collected in Rondônia State). [file peerj-08-9057-s010.docx]

|  | 1. Mean proportion of DNA templates from non-degraded DNA | s.d. | B- Mean proportion of DNA templates from degraded DNA | s.d. |
| --- | --- | --- | --- | --- |
| *Aedeomyia squamipennis ** | 0.127 | 0.020 | 0.09557 | 0.00805 |
| *Aedes* sp. | 0.147 | 0.014 | 0.09495 | 0.01634 |
| *Anopheles* (*Nyssorhynchus*) sp. * | 0.070 | 0.015 | 0.09211 | 0.02556 |
| *Chagasia* sp. | 0.009 | 0.001 | 0.00002 | 0.00003 |
| *Coquillettidia venezuelensis* | 0.040 | 0.004 | 0.05702 | 0.01446 |
| *Culex* (*Culex*) sp. * | 0.013 | 0.002 | 0.01251 | 0.00739 |
| *Culex* sp. | 0.004 | 0.002 | 0.00401 | 0.00251 |
| *Haemagogus* sp. | 0.009 | 0.001 | 0.01555 | 0.00804 |
| *Anopheles* (*Kerteszia*) sp. | 0.004 | 0.001 | 0.01976 | 0.01106 |
| *Limatus* sp. | 0.049 | 0.003 | 0.07138 | 0.02172 |
| *Mansonia flaveola ** | 0.311 | 0.030 | 0.29236 | 0.00559 |
| *Mansonia humeralis ** | 0.014 | 0.001 | 0.00612 | 0.00502 |
| *Mansonia indubitans ** | 0.027 | 0.002 | 0.02879 | 0.00731 |
| *Mansonia titillans ** | 0.136 | 0.018 | 0.15812 | 0.01313 |
| *Psorophora* sp. | 0.002 | 0.001 | 0.00068 | 0.00073 |
| *Wyeomyia* (*Phoniomyia*) sp. | 0.030 | 0.004 | 0.05107 | 0.01137 |
| *Wyeomyia* sp. | 0.008 | 0.001 | 0.09557 | 0.00805 |
